# Critical appraisal of the literature

**DOI:** 10.1590/0100-6991e-20213032

**Published:** 2021-04-24

**Authors:** ÁLIDA ROSÁRIA SILVA FERREIRA, GABRIELA DE OLIVEIRA, BERNARDO FARIA LEVINDO COELHO, MARIA ISABEL TOULSON DAVISSON CORREIA

**Affiliations:** 1 - Universidade Federal de Minas Gerais, Estatística/Demografia/Nutrição - Belo Horizonte - MG - Brasil; 2 - Universidade Federal de Minas Gerais, Escola de Medicina - Belo Horizonte - MG - Brasil; 3 - Universidade Federal de Minas Gerais, Departamento de Cirurgia - Belo Horizonte - MG - Brasil

In the article “Doctor, how long should I isolate?” published in the New England Journal of Medicine on March 10, 2021, two experts (Valeria Fabre and Richard Wenzel) defend different points of view on the duration of isolation for a 24-year-old patient, without comorbidities and with COVID-19. However, she required hospitalization in the Intensive Care Unit, having evolved without complications, but as she lives with her parents, she was considered to be a high risk for disease spreading. Therefore, it is essential to determine the appropriate period of isolation^1^. The reader are stimulated to choose between recommending extended isolation for 20 days or reassure the patient on the low risk of transmission. To support the discussion, there are a total of eight scientific articles published between 2020 and 2021 (a meta-analysis and seven observational studies). However, when analyzing them individually, the question arises: is there enough evidence in each of these articles to justify any of the reader’s choice?

The meta-analysis goal is to characterize the dynamics of viral load, the release duration of viral RNA and the release of viable SARS-CoV-2 viruses in various body fluids, in addition to comparing the viral dynamics of SARS-CoV- 2, SARS-CoV and MERS-CoV2. Among the conclusions, the authors find that the release of SARS-CoV-2 RNA in respiratory and stool samples can be prolonged, but the duration of viral viability is reduced. Furthermore, it is said that the SARS-CoV-2 titers in the upper respiratory tract reach their peak in the first week of the disease^2^.

The interpretation of such results should be done with caution, since the heterogeneity of the selected studies and the fact that all patients have received a variety of treatments are important limitations. Meta-analysis with these flaws are common, and compromise the conclusions. On the other hand, the observational articles that support the decisions of the two specialists also have important limitations, such as the lack of sample calculation and, some of them, lack of representativeness in the composition of the sample^3^ ^,^ ^4^. Without the discussion on sample power, confidence interval and effect size, doubts remain about the ability of these studies to have the results extrapolated to other populations. In fact, Sample size insufficiency threatens the validity and generalizability of any study results. The size of a sample influences two statistical properties: 1) the precision of estimates and 2) the power of the study to draw conclusions. 

Valeria Fabre quotes the viability of the virus in patients with persistently positive SARS-CoV-2 CRP tests in upper respiratory tract samples collected after the first week of the positive CRP test, with viability being observed through in vitro growth in cell cultures^1^ ^,^ ^5^. The study shows the correlation between the CRP cycle threshold values for the diagnosis of SARS-CoV-2 and the growth of the virus in cell cultures, under the context of prolonged viral RNA release, investigating rates of false negative results. However, the interpretation of the data also requires caution, as factors such as the choice of the used cell line to assess viral isolation, the sample size without specification of representativeness and the over-representation of hospitalized patients with various comorbidities can cause errors in the analysis^5^. 

In the case of immunosuppressed patients, the analysis of only 20 subjects with diagnoses of various types of cancer, undergoing different treatments and who had COVID-19, will be enough to allow the inference that the elimination of viable SARS-CoV-2 occurs in two months6? Using the patient availability feature to infer that the sample is sufficient is not supported from a statistical point of view. It is always possible to perform at least a posteriori calculation to understand the size effect and the power of the results.

The careful observation of the studies must be unrestricted and, the example of the study with 129 serious cases of COVID-19, which indicated the probability of about 5% of detection of viable viruses 15 days after the onset of symptoms also has limitations. Sample collection was carried out in a non-predefined time and the fact that the study was a clinical cohort, may have resulted in biased sample collection^7^.

The cross-sectional study that aimed to determine the relationship between the RT-CRP cycle threshold values of the E SARS-CoV-2 RT-CRP gene in respiratory samples, and the onset of symptoms for testing and infectivity of the virus in culture cells, indicated that there was no infectivity at the cycle threshold greater than 24 or onset of symptoms greater than eight days. However, the use of only one SARS-CoV-2 genetic target for analysis, the potential memory bias on the onset of symptoms and the lack of specification as to the severity of each patient are examples of limitations of this study^8^.

Richard Wenzel mentions that the attack rate, in a prospective case study, was 0% for contacts who were exposed to individuals with COVID-19 who had manifested symptoms for more than five days^1^ ^,^ ^9^. The purpose of the latter study was to outline the dynamics of viral transmission and assess the risk of transmission in different periods of time, before and after the onset of symptoms. The researchers concluded that there is high transmissibility before and immediately after the onset of symptoms, (within five days). Among the limitations of this study, there is the fact that the researchers did not fully examine the contacts before the manifestation of the clinical symptoms of index cases, which may have underestimated the importance of early transmission^9^.

All these questions put together with the dissemination of evidence-based health practices (EBP) raise attention to an important question: what is the ideal design of a study to answer a certain clinical question?

In general, different questions must be answered with different study designs. In the NEJM article^1^, the arguments for and against the isolation of the patient were based on evidence from different types of studies. This leads to the following question: can the same question be answered in different ways?

The answer is yes, but apparently, considering the heterogeneity of the presented methods, it is suggested that there are doubts about the adequate “choice” of the references. After all, what would be the ideal way to answer the raised clinical question? In this regard, it is important to highlight a concern “who does not know what is looking for, does not understand what finds”. In view of the earlier mentioned weaknesses, all the assertive recommendations are, to say the least, worrying.

Another important discussion is about the “evidence pyramid”. Here, careful analysis is required: study designs at the “top” of the pyramid should not necessarily be viewed as “better” when compared to other studies. This depends on which clinical question the researcher wants to answer. For example: if the objective is to evaluate the effectiveness of an intervention, randomized clinical trials (RCTs) are the best design; but if, on the other hand, the objective is to follow the evolution of a group over time, a prospective study may be the appropriate option. Furthermore, maintaining systematic reviews and meta-analysis at the top of this pyramid may not be appropriate considering that they are secondary sources of data and should be used to characterize / analyze the original studies as proposed by Murad et al. (2016)^10^. Expecting this type of study to bring different results than the originals and always considering them the “top” of the evidence can be catastrophic for clinical practice.

Regarding the impact factor (IF), a concept that is considered relevant to evaluate a study, it is based on the average number of citations that a journal received over time^11^. Although it is widely used to indicate the quality, the impact factor can’t be used as a validation of the methodological choices and the importance of the study^12^. After all, the number of citations may be influenced by factors such as the research area in which the study is inserted, the number of articles already published on the topic and the relevance of the findings, which can, for example, be readily incorporated into books and reduce the citations of the article^12^. Still, the lack of research ethics and the incentive to academic productivism also make the IF interpretation problematic^12^ ^-^ ^14^. Thus, it is essential to avoid the “glamorization” bias of the IF, that is, the careful analysis must be valued at the expense of the superficial examination of articles due to the high factor of the journal in which they are published.

It is extremely important to emphasize that some health professionals do not have adequate knowledge about the scientific method, impairing the critical analysis of published information^3^ ^,^ ^15^ ^,^ ^16^. In addition, most studies may have false results, considering aspects such as statistical power, bias and level of significance. In studies with reduced sample size, for example, there may be less statistical power, resulting in a decrease in the positive predictive value^17^.

It is well known that in the context of a pandemic, there is a need to quickly obtain data to develop strategies to face the unfavorable scenario. However, this should be seen with caution as it can damage the design of the studies and, consequently, cause misinterpretation of results, which negatively impacts the adoption of conducts that, in turn, interfere with better patient care^4^ ^,^ ^15^.

Therefore, together with the notoriety of clinical reasoning and ethical principles in the practice of the healthcare profession, it is essential that there is greater critical sense and knowledge about scientific methods in order to properly guide patients and promote better quality of care^4^.
